# AMRrounds: presumed mechanisms of resistance in a case of XDR *Klebsiella pneumoniae* empyema

**DOI:** 10.1093/jacamr/dlaf019

**Published:** 2025-02-20

**Authors:** Michael Casias, Madison Salam

**Affiliations:** Department of Pharmacy, University of Colorado Hospital, 12605 E. 16th Ave., Aurora, CO, USA; Department of Pharmacy, University of Colorado Hospital, 12605 E. 16th Ave., Aurora, CO, USA

## Case

**Figure 1.**
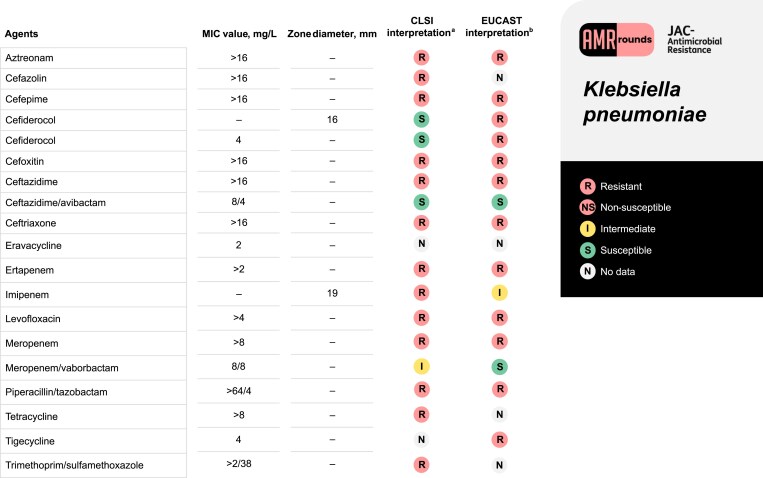
Antimicrobial susceptibility profile for the *K. pneumoniae* isolate corresponding to the clinical case. ^a^MIC testing was performed using BD Phoenix Gram-negative MIC309 panel (BD Diagnostic Systems, Sparks, MD, USA). Exceptions include eravacycline, which was performed using an ETEST (bioMérieux), imipenem and cefiderocol, which were performed using Kirby–Bauer disc diffusion, and cefiderocol confirmatory susceptibilities which were determined using broth microdilution from a reference laboratory. CLSI M100-ED33:2023 version was used for interpretation and reporting of susceptibility results. ^b^EUCAST Clinical Breakpoint Tables v. 13.1, 2023 was also used for comparison for susceptibility results.

A 75-year-old man (weight 86.2 kg) was referred for tertiary care at our institution after a chest, abdomen and pelvic CT scan revealed a complex right pleural effusion with a percutaneous biliary drain traversing the pleural sulcus that was concerning for a pleural–biliary fistula. His past medical history was significant for cirrhosis secondary to primary sclerosing cholangitis that was complicated by recurrent cholangitis, chronic biloma with biliary drain, and multiple hospitalizations due to *Klebsiella pneumoniae* complex (KpnC) bacteraemia that had previously been treated with two 6 week courses of ceftriaxone followed by meropenem. Upon examination, he was afebrile with a temperature of 36.2°C (97.2°F), blood pressure of 108/74 mmHg, and pulse of 94 beats/min. He was breathing at 16 breaths/min and oxygen saturation was 90% on ambient air. His WBC count was 11.6 × 10^9^ cells/L. Lung findings on physical exam were unremarkable. His abdomen was tender with constipation noted. A thoracentesis was performed, demonstrating 6618 × 10^6^/L nucleated cells with 85% neutrophils, pleural fluid protein 3.8 g/dL and glucose <10 mg/dL. His pleural fluid grew an MDR *K. pneumoniae* (see Figure 1). The patient was originally on meropenem 1 g IV every 8 h (infused over 3 h) but was transitioned to ceftazidime/avibactam 2.5 g every 8 h (infused over 3 h).

What is the most likely explanation for this resistance profile and recommendation for treatment?

## Discussion

Isolates in the KpnC are encapsulated, Gram-negative commensal organisms capable of producing an MDR or XDR phenotype. After receiving this culture, identity was confirmed via MALDI-TOF and susceptibility testing was performed using BD Phoenix^™^ according to CLSI guidance.

KpnC isolates are intrinsically resistant to penicillins and aminopenicillins, due to a chromosomal SHV-1 β-lactamase (Table 1).^1^ Hyperexpression of this enzyme can confer resistance to ceftazidime and can overwhelm inhibitors such as tazobactam.^1^ Outside of their intrinsic resistance, KpnC isolates can acquire broader β-lactamases via plasmids, including ESBL genes such as *bla*_CTX-M_.^2^ These ESBL-encoding plasmids may also co-harbour aminoglycoside-modifying enzyme genes, notably *AAC(6*′*),* rendering aminoglycosides ineffective.^3^ Plasmids may also contain AmpC-encoding genes such as *bla*_DHA-1_ and *bla*_CMY-2_.^4^ It is difficult to discern the genetic basis for a phenotype without sequencing; however, the presence of ceftriaxone, cefepime and aminoglycoside resistance suggest a plasmid containing one of these β-lactamase genes, likely ESBL and *AAC(6*′*)*, was present in the isolate at hand.

**Table 1.** Intrinsic and acquired resistance mechanisms reported for *K. pneumoniae*

Resistance mechanism	Intrinsic versus acquired resistance	Antimicrobials affected, resulting in resistance
OmpK35/36 porin loss	Acquired	Cefazolin, cefoxitin, cefepime, meropenem
SHV-1 β-lactamase	Intrinsic	Penicillins, aminopenicillins
Class A β-lactamase, ESBL and KPC		
*bla*_CTX-M-15_	Acquired	Penicillins, cephalosporins
*bla*_KPC_	Acquired	Penicillins, cephalosporins, carbapenems
Class B β-lactamase, MBL, carbapenemase		
*bla*_NDM-1_	Acquired	Penicillins, cephalosporins, carbapenems
Class D β-lactamase, oxacillinase		
*bla*_OXA-48-like_	Acquired	Penicillins, cephalosporins (except ceftazidime/avibactam and cefiderocol), carbapenems
Aminoglycoside-modifying enzyme		
*aac(6*′*)*	Acquired	Aminoglycosides
RND-type efflux pump		
AcrAB-TolC	Acquired	Tetracyclines, glycylcyclines, fluoroquinolones, cefoxitin

Though KpnC isolates can acquire carbapenemase genes, specifically *bla*_KPC_ in the USA, this isolate was carbapenemase negative by Cepheid Xpert Carba-R^®^ (*bla*_KPC_, *bla*_VIM_, *bla*_OXA-48-like_, *bla*_IMP-1_, *bla*_NDM_) PCR.^5^ Indeed, non-carbapenemase (non-CP) carbapenem resistance is well described in *K. pneumoniae*.^5–7^ A 2020 US study found that 39% of their carbapenem-resistant (CR) *K. pneumoniae* harboured no carbapenemase, and another 15% remained unconfirmed.^5^ It appears that the combination of porin loss (OmpK35 > OmpK36) plus a β-lactamase, specifically CTX-M-15 and AmpC-type enzymes, can produce phenotypic carbapenem resistance.^5^ In another US-based sample of non-CP CR-*K. pneumoniae*, all organisms carried a CTX-M-type organism, and 91% had porin mutations.^7^

KpnC isolates rely heavily on outer membrane porins, most notably OmpK35/36.^2^ Porin loss is associated with varying degrees of resistance.^2^ In a knockout model evaluating the loss of OmpK35/36 from WT *K. pneumoniae*, MIC increases varied widely by substrate. Of particular interest to this case, knockout mutants displayed an 8-fold increase in meropenem MICs. In knockout mutants that also harboured CTX-M-14 or CMY-12, meropenem MICs increased from 0.06 to 1 and 2 mg/L, respectively. Comparatively, ceftazidime MICs in knockout mutants only increased 2-fold.^2^ This may explain ceftazidime/avibactam susceptibility in our isolate: ceftazidime’s decreased dependence on porins combined with avibactam’s robust activity against Ambler Class A enzymes including CTX-M-15. Meropenem/vaborbactam, by contrast, relies more on porin channels for meropenem’s entrance into the bacterial cell.

Efflux pumps also contribute to KpnC’s robust resistance artillery. AcrAB-TolC, a resistance-nodulation-division (RND) efflux pump, is commonly implicated in drug resistance.^8^ Insertion mutations in AcrAB-TolC’s regulator gene, *acrR*, and overexpression of its activator gene, *ramA*, are associated with overexpression of *acrB* that encodes for AcrAB-TolC itself. Efflux pump overexpression has been documented to confer resistance to tetracyclines and glycylcyclines, fluoroquinolones and cefoxitin, yet leaves carbapenem MICs unaffected.^8^ This would neatly explain the non-susceptibility to fluoroquinolones, tetracyclines and tigecycline in our isolate.

Cefiderocol is ostensibly well poised for success here given its stability in the face of outer membrane porin mutations and serine β-lactamases. In an *in vitro* study of MDR Enterobacterales with ESBL plus porin loss (most likely OmpK35), however, only 61.5% (16/26) of isolates were cefiderocol susceptible (MIC ≤ 2 mg/L). Addition of avibactam to cefiderocol did potentiate its effects, suggesting some degradation by β-lactamases.^9^ Given that the most likely theory of this KpnC isolate’s phenotype includes an ESBL plus porin loss, it is unsurprising that cefiderocol was non-susceptible.

### Conclusions

In this specific isolate, the XDR phenotype appears to be due to a combination of porin loss, the production of a chromosomal SHV-1 β-lactamase, an acquired plasmid containing genes encoding for ESBL or AmpC enzymes as well as an aminoglycoside-modifying enzyme, and efflux pump overexpression. This would account for resistance to β-lactam and monobactam antimicrobials, tetracyclines and glycylcyclines, fluoroquinolones and aminoglycosides. The patient ultimately underwent a pigtail drain, removal of biliary drain, placement of common bile duct stent and embolization of the tract, and succeeded on standard dosing of ceftazidime/avibactam for 6 weeks.
