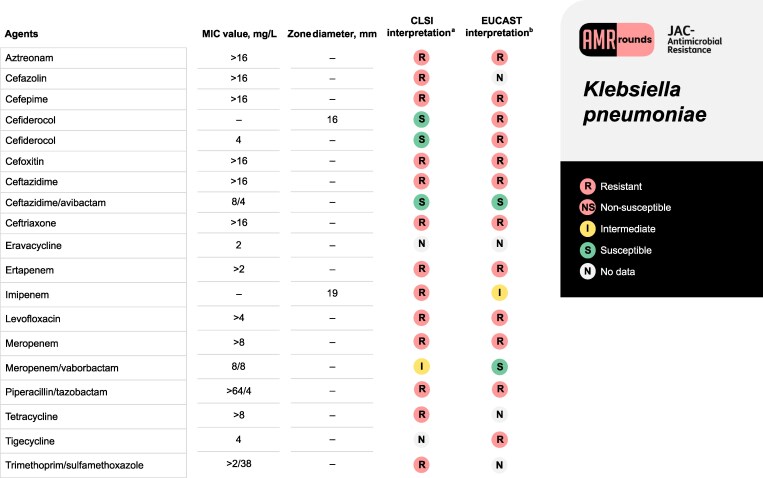# Correction to: AMRrounds: presumed mechanisms of resistance in a case of XDR *Klebsiella pneumoniae* empyema

**DOI:** 10.1093/jacamr/dlaf104

**Published:** 2025-06-12

This is a correction to: Michael Casias, Madison Salam, AMRrounds: presumed mechanisms of resistance in a case of XDR *Klebsiella pneumoniae* empyema, *JAC-Antimicrobial Resistance*, Volume 7, Issue 1, February 2025, dlaf019, https://doi.org/10.1093/jacamr/dlaf019

In the originally published online version of this manuscript, the following susceptibility interpretations were made in error:

- Imipenem with a zone diameter of 19 mm was interpreted as resistant using the EUCAST Clinical Breakpoint Tables v. 13.1, 2023. However, since there is no area of technical uncertainty, this should have been interpreted as “intermediate.”
- Trimethoprim/sulfamethoxazole with a minimum inhibitory concentration (MIC) of >2/38 mg/L was interpreted as susceptible. However, due to the unknown MIC, it should have been interpreted as “no data.”
- Eravacycline had an interpretation for its respective MIC’s, however since CLSI M100 does not provide susceptibility interpretation, it should’ve been interpreted as “no data.” Additionally, since EUCAST has interpretation breakpoints for only *E. coli,* it should’ve been interpreted as “no data.”
- Cefiderocol with a zone diameter of 16 mm was interpreted as intermediate but should have been interpreted as “susceptible”
- Tigecycline had an interpretation for its respective MIC, however since CLSI M100 does not provide susceptibility interpretation, it should’ve been interpreted as “no data.”
- Cefoxitin (>16 mg/L) and tetracycline (>8 mg/L) MIC’s were interpreted as “resistant” since the assumption is the next dilution would be categorized as resistant.

All changes have been corrected in Figure 1

**Figure:**